# DEVELOPING AND TESTING A TWO-PART INTERVENTION: ENHANCING DEMENTIA INSTRUCTION AND TOOL IN HOME HOSPICE CARE

**DOI:** 10.1093/geroni/igad104.2650

**Published:** 2023-12-21

**Authors:** Sophia Geisser, Abigail Aamponsah, Felix Vasquez, Elizabeth Luth

**Affiliations:** The University of Alabama, Tuscaloosa, Alabama, United States; Rutgers, The State University of New Jersey, New Brunswick, New Jersey, United States; VNS Health, New York City, New York, United States; Rutgers University, New Brunswick, New Jersey, United States

## Abstract

Family care partners of persons living with dementia provide extensive care and experience significant stress and strain, particularly near the person living with dementia’s end of life. Hospice nurses and social workers (clinicians) are positioned to support family care partners, but seldom receive training to do so. This article describes the approach to developing and pilot testing a two-part intervention, Enhancing Dementia Instruction and Tool in Home Hospice Care (EDITH-HC). This intervention provides 1) educational videos for hospice nurses and social workers about dementia-specific end-of-life care and 2) a worksheet for clinicians to use as part of their clinical practice to use with family care partners to identify and address their stressors and concerns. EDITH-HC development involved: (1) a systematic review of literature and analysis of interview data from 58 family care partners (n=40) of home hospice patients living with dementia and clinicians (physicians, nurses, and social workers, n=18); (2) analysis of primary data collected from two rounds of structured, key stakeholder input from 19 family care partners, hospice clinicians, and research/content experts; and (3) single-arm pilot of the intervention to test initial feasibility and acceptability. The intervention development will lead to a randomized pilot study to determine the feasibility and acceptability of implementing the training and worksheet in clinical practice compared to usual care.

